# Sex Differences in the Cardiovascular Significance of Albuminuria in Type 2 Diabetes: A Narrative Review

**DOI:** 10.3390/jcm15176913

**Published:** 2026-09-07

**Authors:** Carlos Enrique Martínez-Alberto, Zuelika Bobadilla-Hernández, Javier Donate-Correa

**Affiliations:** 1 Escuela de Enfermería Nuestra Señora de Candelaria, Hospital Universitario Nuestra Señora de Candelaria, 38010 Santa Cruz de Tenerife, Spain; extcmartina@ull.edu.es; 2 Unidad de Investigación, Hospital Universitario Nuestra Señora de Candelaria, 38010 Santa Cruz de Tenerife, Spain; 3 Instituto de Investigación Sanitaria de Canarias (IISC), 35012 Las Palmas de Gran Canaria, Spain; 4 Centro de Salud San Benito, Servicio Canario de Salud, 38203 San Cristóbal de La Laguna, Spain; zuleika_bauta@yahoo.es; 5 Instituto de Tecnologías Biomédicas (ITB), Universidad de La Laguna, 38200 San Cristóbal de La Laguna, Spain; 6 GEENDIAB (Grupo Español Para el Estudio de la Nefropatía Diabética), Sociedad Española de Nefrología, 39000 Santander, Spain; 7 RICORS2040 (Redes de Investigación Cooperativa Orientadas a Resultados en Salud)-Renal (RD24/004/0022), Instituto de Salud Carlos III, 28000 Madrid, Spain

**Keywords:** albuminuria, urinary albumin-to-creatinine ratio, sex differences, type 2 diabetes, cardiovascular risk, diabetic kidney disease, A1 albuminuria

## Abstract

Albuminuria is routinely used to assess kidney damage in diabetes, but it also conveys cardiovascular risk across the urinary albumin-to-creatinine ratio (UACR) distribution, including within A1. Whether sex modifies this relationship remains uncertain. This narrative review prioritizes longitudinal studies in type 2 diabetes mellitus (T2DM), analyses within A1 particularly, and distinguishes this direct evidence from the general population or chronic kidney disease cohorts, surrogate-endpoint studies, and experimental mechanisms. A few studies report formal sex-by-UACR interactions for selected outcomes, including mortality in broad kidney-disease populations and heart failure in diabetes. Many other apparent differences derive only from sex-stratified analyses and therefore do not establish effect modification. Differences in urinary creatinine excretion, body composition, hormonal exposure, kidney injury pathways, cardiovascular phenotype, and health-care ascertainment may alter the observed association, but most mechanistic evidence is indirect. Current evidence supports UACR as a continuous prognostic marker in both sexes but does not justify sex-specific diagnostic cut-offs, therapeutic thresholds, or response targets. UACR should be measured systematically, confirmed when abnormal or borderline, preferably repeated under standardized conditions, and interpreted with eGFR and the overall cardiovascular risk profile. Sex may provide additional clinical context; treatment should continue to follow guideline-based indications.

## 1. Introduction

Chronic kidney disease (CKD) attributed to diabetes affects approximately 20–40% of people with diabetes and is associated with substantial cardiovascular risk [1,2]. Current KDIGO classification defines normal to mildly increased albuminuria (A1) as UACR < 30 mg/g, moderately increased albuminuria (A2) as 30–299 mg/g, and severely increased albuminuria (A3) as ≥300 mg/g [1,2]. These categories support staging and treatment decisions, but cardiovascular risk, CKD progression, and mortality increase continuously across the UACR distribution rather than changing abruptly at 30 or 300 mg/g [2,3].

A1 does not imply absence of kidney or cardiovascular risk. eGFR may decline while UACR remains <30 mg/g, and cohort, trial, and meta-analytic data show graded associations between UACR within A1 and cardiovascular events or mortality [4,5,6,7,8]. This distinction is central: UACR can be a continuous prognostic marker without every value within A1 constituting kidney disease or a treatment indication.

The less established question is whether the same UACR carries the same prognostic information in women and men. Meta-analyses show that diabetes is associated with a greater relative excess risk of coronary and other cardiovascular outcomes in women than in men [9,10]. That observation provides a rationale for examining effect modification, but it does not identify albuminuria as the mediator or demonstrate that the UACR-risk association differs by sex. In this review, sex refers to biological attributes, including anatomy, hormones, chromosomes, and sex-related body composition; gender refers to social roles, behaviors, identities, and health-system processes. Diabetes and kidney disease have sex-related biological contexts, whereas differences in recognition, testing, and treatment may reflect gender-related or structural influences [9,10,11,12,13,14].

UACR also has sex-related measurement constraints. Urinary creatinine excretion is generally lower in women, so the same absolute urinary albumin excretion can yield a higher ratio [15]. Age, muscle mass, body composition, and hormonal status add variation. Experimental evidence indicates sex-related differences in podocyte integrity, tubular metabolism, oxidative and inflammatory signaling, and intrarenal hemodynamics [12,16,17,18]. These mechanisms may alter the context in which albuminuria develops, but they do not establish different cardiovascular thresholds.

This narrative review therefore asks whether sex modifies the association between albuminuria and cardiovascular risk, prioritizing longitudinal evidence in T2DM and UACR within A1. It first establishes the evidence for UACR as a continuous risk marker, then evaluates studies that directly compare its prognostic association in women and men. Evidence from the general population, non-diabetic CKD, T1DM, surrogate cardiovascular phenotypes, and experimental models is used only to assess external consistency, measurement effects, or biological plausibility and is identified as indirect evidence. The clinical aim is to distinguish a reproducible sex interaction from descriptive subgroup differences and to determine whether any such interaction could justify different risk interpretation or treatment decisions.

## 2. Methods

This narrative review was informed by structured searches of PubMed/MEDLINE and Europe PMC from database inception through 31 August 2026. Search concepts combined terms for albuminuria (albuminuria, urinary albumin excretion, UACR, albumin-to-creatinine ratio, low-grade albuminuria, A1), sex or gender (sex, gender, women, men, female, male, menopause), diabetes (diabetes, T2DM, T1DM, diabetic kidney disease), and cardiovascular outcomes (cardiovascular disease, mortality, major adverse cardiovascular events, coronary disease, stroke, heart failure, arterial stiffness, myocardial fibrosis). Targeted searches addressed urinary creatinine, body composition, sampling variability, kidney mechanisms, health-care testing, and sex-specific treatment response. Reference lists and citing articles of key studies and reviews were also screened.

We prioritized meta-analyses and longitudinal human studies reporting hard cardiovascular outcomes or mortality, followed by diabetes-specific cohorts and analyses restricted to A1. To evaluate effect modification, studies had to report sex-stratified estimates or a formal sex-by-UACR interaction; these two forms of analysis were recorded separately. General population or non-diabetic CKD cohorts were retained as indirect epidemiological evidence. Cross-sectional studies and studies of arterial stiffness, myocardial fibrosis, or other surrogate phenotypes were included only when they informed phenotype-specific plausibility. Experimental and mechanistic studies were restricted to work directly comparing females and males or testing sex-hormone or sex-linked pathways and were treated as indirect for the clinical prognostic question.

Study selection was purposive rather than systematic: relevance to the central question, design, population, UACR range, outcome type, and availability of sex-specific or interaction analyses guided inclusion. Randomized-trial and post hoc treatment analyses were included only when they addressed sex-specific cardiorenal effects or albuminuria response. No formal risk-of-bias instrument, duplicate screening, or exhaustive study-identification process was used; these limitations are consistent with the narrative-review design.

## 3. Albuminuria as a Continuous Cardiovascular Risk Marker in Diabetes

The conventional 30 mg/g threshold was developed primarily to identify kidney damage and risk of diabetic nephropathy, not the level at which cardiovascular risk begins [1,2]. In HOPE, among 9043 high-risk adults including 3498 with diabetes, cardiovascular events increased progressively across the UACR distribution even after participants with microalbuminuria were excluded (UACR < 2 mg/mmol, approximately <18 mg/g) [19]. UKPDS likewise showed progressively higher cardiovascular mortality across worsening kidney phenotypes [20]. These studies established a graded association, but neither was designed to determine whether its slope differed by sex.

Subsequent studies established that albuminuria and filtration provide complementary rather than interchangeable prognostic information. In 10,640 participants with T2DM in ADVANCE, higher UACR and lower eGFR independently predicted cardiovascular and renal outcomes; after correction for regression dilution, a tenfold higher UACR was associated with a 2.48-fold higher cardiovascular risk [21]. An individual-participant meta-analysis including more than one million adults, over 128,000 of whom had diabetes, confirmed associations of albuminuria with mortality and kidney failure [22]. Pooled CANVAS/CREDENCE data and large longitudinal T2DM cohorts further linked higher UACR to heart failure, cardiovascular events, mortality, or kidney outcomes within eGFR strata [23,24,25,26,27]. This is strong evidence for prognostic value, although not all analyses were restricted to A1 or designed to assess sex interaction.

Within A1, BENEDICT followed 1208 participants with T2DM for a median of 9.2 years and identified a continuous, nonlinear association between measurable albuminuria and cardiovascular events without an evident threshold [7]. ACCORD/ACCORDION likewise found that UACR predicted MACE and mortality within A1 over a median of 8.83 years [8]. LEADER and SAVOR-TIMI 53 showed progressively higher risk across low UACR categories, while a systematic review and meta-analysis associated values of 10–30 mg/g with higher mortality and coronary risk than very low UACR [6,28,29]. RIACE analyses provide additional T2DM evidence [30]. The incremental value of UACR may nevertheless be attenuated after contemporary cardiac biomarkers are included [29]. Thus, A1 spans a range of risk; it should not be relabeled as a disease category on the basis of a single low-grade value.

UACR is also dynamic. Early reductions in LEADER and EMPA-REG OUTCOME were associated with lower subsequent cardiorenal risk, but post hoc associations do not prove that albuminuria itself is the causal mediator of benefit [31,32]. In DCCT/EDIC, remission of microalbuminuria did not fully normalize cardiovascular prognosis [33]. A falling value may therefore reflect improvement in current vascular-kidney stress without erasing information carried by previous or cumulative albuminuric exposure. Repeated measurements and longitudinal trajectories may be more informative than one baseline or follow-up sample.

The practical distinction is between a continuous prognostic marker and a validated diagnostic or therapeutic threshold. UACR below 30 mg/g can refine risk assessment, especially when persistent, but it does not by itself establish CKD or identify a treatment whose benefit has been demonstrated at that value. Interpretation still requires eGFR, the broader kidney phenotype, cardiovascular status, competing biomarkers, and trial-based treatment indications.

## 4. Clinical Evidence for Sex Differences in Albuminuria-Associated Cardiovascular Risk

This section applies an explicit evidence hierarchy. Greatest weight is given to longitudinal diabetes cohorts with hard cardiovascular outcomes and formal sex-by-UACR interaction testing. The general population or CKD cohorts are indirect for T2DM, and cross-sectional or surrogate-endpoint studies provide only supportive context. A statistically significant association in one sex and a non-significant association in the other does not demonstrate effect modification; the estimates must be compared through a formal interaction test. Table 1 records these distinctions.

The strongest broad epidemiological evidence for effect modification comes from the CKD Prognosis Consortium. In an individual-participant meta-analysis of 46 cohorts including more than two million adults, albuminuria predicted all-cause and cardiovascular mortality in both sexes, while the relative mortality gradient was steeper in women and the interaction by sex was significant [34]. This large meta-analysis carries substantial weight for external consistency but is not diabetes-specific, and a relative interaction does not automatically imply different absolute risks or clinically useful thresholds. The smaller Rancho Bernardo Study reported mortality associations in older women, but not clearly in men; without a direct comparison of estimates, this stratified pattern is hypothesis-generating rather than proof of interaction [41].

Population-based evidence is not uniform. In HUNT-2, 9158 participants provided three urine samples and were followed for 14 years. Albuminuria predicted myocardial infarction or coronary death with similar strength in women and men, and the formal comparison was not significant [35]. Repeated sampling improved classification at low UACR and was particularly relevant because within-person variability was greater in women. This study illustrates that better exposure measurement can narrow apparent subgroup differences rather than reveal a stronger association in one sex.

Small diabetes-specific studies provide limited evidence. Zandbergen et al. followed 67 people with T2DM, microalbuminuria, and no established CVD; women had more macrovascular events, but because every participant had microalbuminuria, the study assessed vulnerability within an albuminuric phenotype rather than a sex difference in the UACR-risk slope [42]. Nakhjavani et al. found an association between albuminuria and ischemic heart disease in men but not women in a matched case–control study [43]. Neither stratified result alone establishes interaction, and both are constrained by sample size, design, and endpoint definition.

The Hoorn Diabetes Care System cohort provides stronger diabetes-specific evidence that any modification may be outcome-dependent. Among 13,657 people with diabetes (98.9% T2DM) followed for a mean of 7 years, sex significantly modified the association between UACR and incident HF (*p* for interaction = 0.010) [36]. UACR > 3 mg/mmol (approximately >27 mg/g) was associated with higher HF risk in women but not men. Because this category includes the upper end of A1 and values above it, the finding supports an interaction hypothesis for HF but is not evidence confined to low-grade albuminuria within A1. Other cardiovascular endpoints did not show the same pattern.

Siverio-Morales et al. provided direct evidence within A1. In 420 adults with T2DM, preserved kidney function, and UACR < 30 mg/g, higher UACR was associated with greater coronary stenosis and MACE over 6 years [37]. Estimates were directionally stronger in women, but the UACR-by-sex interaction was not statistically significant. The study therefore supports the prognostic value of UACR within A1 in both sexes; it does not demonstrate a true difference between them.

Xuan et al. found an association between normal-range UACR and baPWV in women but not men among 1119 adults with T2DM [38]. The cross-sectional design, surrogate vascular endpoint, and absence of a reported formal interaction limit inference. The findings indicate a potentially sex-related vascular phenotype, particularly after menopause, rather than a validated difference in clinical risk.

Indirect studies also show that the direction is not consistently female-predominant. In MESA, higher urinary albumin and UACR were associated with cardiac magnetic-resonance markers of interstitial myocardial fibrosis mainly in men [44]. In 11,701 generally healthy older ASPREE participants followed for a median of 8.3–8.6 years, low-level UACR predicted mortality in both sexes, whereas CVD, MACE, and stroke associations were more consistently apparent in men [40]. These studies concern different populations and phenotypes and cannot be transferred directly to T2DM within A1.

In a small CKD study, sex modified the UACR-AIx association, with a positive association in women, but did not modify associations with pulse wave velocity or mean arterial pressure [39]. This formal but phenotype-specific interaction involves surrogate vascular measures and remains indirect for clinical cardiovascular outcomes in T2DM.

The most defensible conclusion is that UACR predicts cardiovascular risk in both sexes, whereas evidence that sex modifies this relationship is limited, outcome-specific, and inconsistent. Formal interaction has been shown for selected outcomes, including mortality in broad CKD populations and HF in a diabetes cohort, but direct evidence within A1 has not demonstrated a significant interaction [34,36,37]. Most individual studies were not powered primarily for interaction testing, and differences in point estimates may reflect sampling variation, exposure misclassification, or outcome heterogeneity. Even a reproducible statistical interaction would require external validation and evidence of improved prediction or decision-making before it could support different thresholds. No consistent direction or clinically validated sex-specific UACR threshold emerges from the current evidence.

## 5. Methodological Considerations in the Interpretation of UACR

Interpretation of UACR requires particular caution when sex differences are examined. UACR is a practical surrogate for timed urinary albumin excretion, but the creatinine denominator varies with muscle mass, age, body size, diet, and sex. Two people with identical absolute albumin excretion may therefore have different ratios. This calibration issue is most influential within A1, where small absolute differences can appear biologically meaningful. Table 2 summarizes measurement, biological, health-care, and treatment factors that can alter the observed UACR-cardiovascular association.

Sex-related differences in urinary creatinine excretion have been recognized for decades. Connell et al. found that creatinine excretion was approximately 55% higher in men than in women and that the relationship between albumin excretion rate and UACR differed substantially by sex [15]. Houlihan et al. compared morning spot UACR with 24-h albumin excretion in 314 people with diabetes. For a given albumin excretion rate, UACR was higher in women and increased with age in both sexes, producing more false-positive classifications among older participants [45]. Mattix et al. showed the population-level effect of the same denominator. Although mean urinary albumin concentrations were similar in women and men, a common UACR threshold of 30 mg/g yielded a higher estimated prevalence of microalbuminuria in women (9.2% vs. 6.0%). The difference was no longer significant when sex-specific cut-points were used [46]. This finding demonstrates a calibration effect and does not by itself establish that sex-specific thresholds improve cardiovascular risk prediction.

The creatinine denominator has two related but non-equivalent roles. First, lower creatinine excretion can raise UACR for the same albumin excretion, producing a calibration difference between people with different body composition. Second, low urinary creatinine may itself carry prognostic information related to muscle mass, frailty, or general health. PREVEND and MESA indicate that both urinary albumin and urinary creatinine contribute to cardiovascular associations [47,48]. Conversely, high creatinine generation in severe obesity can yield a normal UACR despite elevated 24-h albumin excretion, particularly in men with greater fat-free mass [49]. A calibration artifact should therefore not be assumed to explain all prognostic information in the denominator.

Urine collection procedures constitute a second major source of variability. Albumin excretion changes with posture, physical activity, hydration, glycemic control, blood pressure, fever, and other transient conditions. Twenty-four-hour UAE avoids dependence on urinary creatinine concentration but is cumbersome and prone to collection error. First-morning samples provide a practical compromise. Witte et al. found that first-morning UACR agreed more closely with 24-h UAE and had lower intraindividual variability than random spot measurements [50]. In a separate population-based analysis, first-morning UACR predicted cardiovascular morbidity and mortality similarly to 24-h UAE [51]. Sampling differences are especially relevant at low UACR, where measurement noise may approach the magnitude of the differences under study.

A single UACR measurement is also limited by biological variability. Rasaratnam et al. obtained four spot measurements within four weeks in 826 people with T2DM and reported a within-individual coefficient of variation of 48.8%. A repeated value could fall between approximately 0.26 and 3.78 times the initial measurement within the 95% limits of random variation [52]. Female sex was independently associated with greater variability [52]. Primary-care data from 773 people with T2DM likewise showed a median coefficient of variation of approximately 41% and substantial misclassification when staging did not incorporate repeated measurements [53]. If variability differs by sex, regression dilution and category misclassification may also differ and distort apparent sex-specific associations.

Repeated sampling therefore matters for both diagnosis and research. Guidelines recommend confirmation of an abnormal result rather than reliance on a single measurement [1,2]. At values below 30 mg/g, averaging repeated first-morning measurements can reduce random variation and provide a more stable estimate of chronic albumin exposure. HUNT-2 illustrates this point. Three urine samples improved cardiovascular risk classification at low albuminuria levels, and within-person variability was greater in women [35]. Studies based on a single sample may thus be less able to detect genuine sex-by-UACR interactions.

A further distinction concerns UACR as a marker of current vascular-kidney stress versus cumulative albuminuric exposure. Albuminuria changes with glycemic control, blood pressure, RAS blockade, SGLT2 inhibitors, GLP-1 receptor agonists, and other therapies. Short-term UACR reductions are associated with improved subsequent cardiorenal outcomes [31,32], but a single follow-up value may not capture the vascular consequences of previous exposure. Longitudinal measures such as time-updated UACR, cumulative exposure, or within-person slopes may therefore provide information that is not available from one baseline measurement.

These considerations complicate sex-specific interpretation. Higher UACR in women may partly reflect lower urinary creatinine excretion rather than greater absolute albumin loss. Conversely, higher creatinine generation in men with greater muscle or fat-free mass can reduce UACR despite elevated absolute albumin excretion [47,49]. Adjusting these differences away entirely could also remove prognostic information related to body composition. Greater variability in women may further weaken associations and reduce power for interaction testing. Neither a common threshold nor sex-specific thresholds can therefore be assumed a priori to optimize risk discrimination. Studies should report sampling protocols, repeated measurements, UACR distributions, and urinary albumin and creatinine components by sex.

## 6. Biological Plausibility and Its Limits

**Figure 1:**
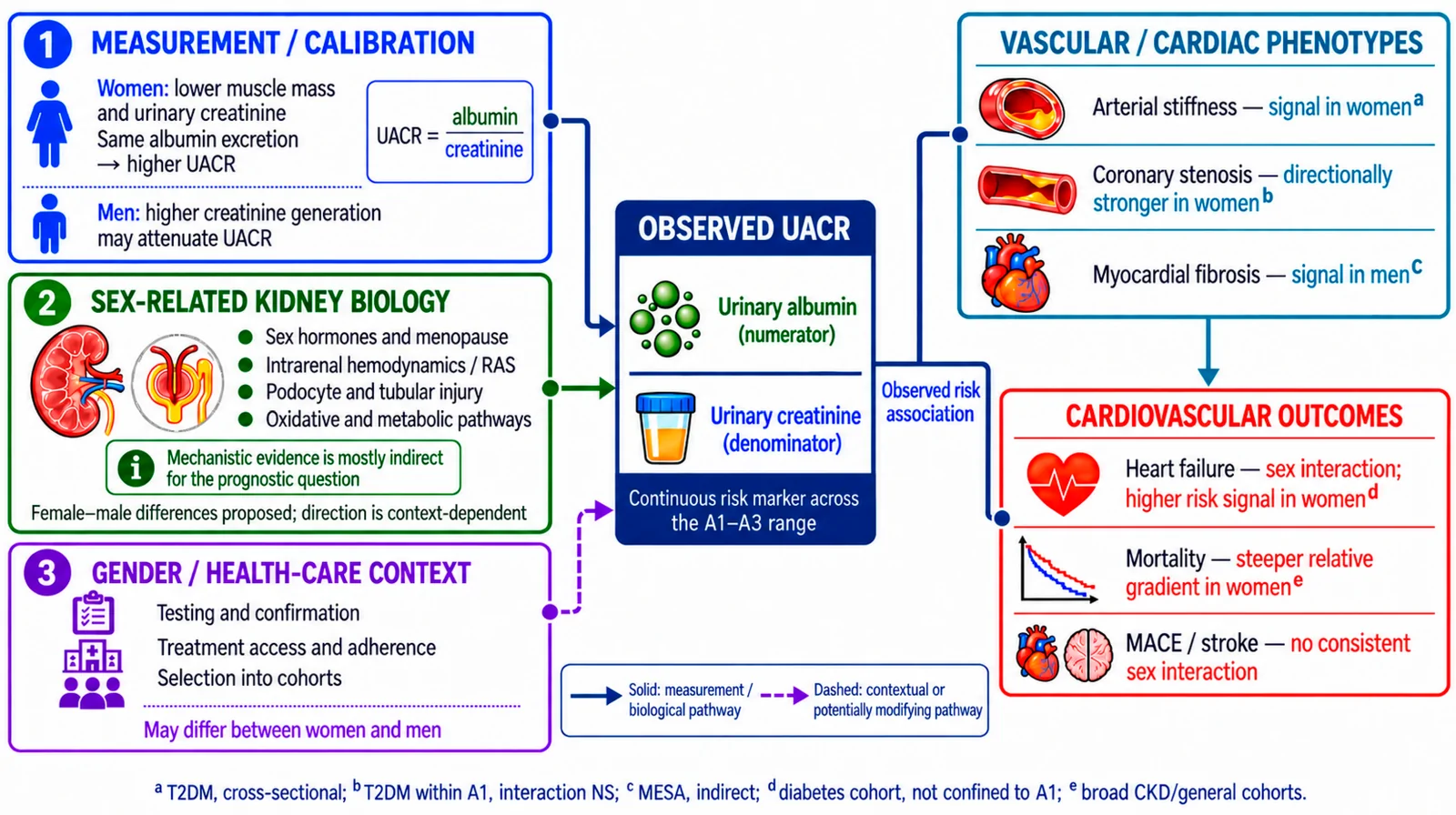
Conceptual framework for sex- and gender-related influences on the measurement and cardiovascular interpretation of UACR. The diagram distinguishes ratio calibration, kidney biology, health-care context, and reported phenotype- or outcome-specific associations. Solid arrows denote measurement or biological pathways; dashed arrows denote contextual or potentially modifying pathways. Evidence qualifiers distinguish direct from indirect findings and formal interactions from directional subgroup patterns. Lettered superscripts (**a**–**e**) identify the specific evidence context underlying each phenotype- or outcome-related statement, as defined at the bottom of the figure. A1, normal to mildly increased albuminuria; CKD, chronic kidney disease; MACE, major adverse cardiovascular events; MESA, Multi-Ethnic Study of Atherosclerosis; RAS, renin–angiotensin system; T2DM, type 2 diabetes mellitus; UACR, urinary albumin-to-creatinine ratio.

Direct mechanistic evidence explaining why the same UACR would carry different cardiovascular information in women and men is lacking. Three related questions should not be conflated: why albuminuria develops, why the measured ratio differs for the same albumin excretion, and why a given UACR might predict a cardiovascular outcome differently. Most mechanistic studies address the first question, whereas body composition and urinary creatinine address calibration of the second question. These pathways can support biological plausibility but cannot establish a clinical sex-by-UACR interaction or justify different thresholds (Figure 1).

Sex steroids and intrarenal hemodynamics may alter the development of albuminuria. Estrogenic and androgenic signaling can influence the renin-angiotensin system, glomerular pressure, endothelial function, and podocyte survival. Experimental work shows opposing effects of testosterone and 17β-estradiol on podocyte apoptosis, severe injury in estrogen-deficient female diabetic mice, and sex-dependent responses to ACE2 deficiency and angiotensin II [54,55,56]. Menopause may change this hormonal context, but age is an imprecise surrogate for reproductive status. Cross-sectional human data link circulating sex hormones to UACR or diabetic kidney disease in men and postmenopausal women; these associations are compatible with biological relevance but cannot establish direction or causality [57].

Tubular pathways provide complementary plausibility. Translational work using human proximal tubular cells identified donor-sex, androgen, metabolic, and X-linked epigenetic effects on cellular stress responses [18]. However, cell systems and small donor samples do not reproduce the integrated hemodynamic and cardiovascular setting of T2DM. Female mice showed a marked early medullary NOX4 response in experimental T1DM, while estradiol-dependent preservation of tubular AMP-activated protein kinase signaling has been linked to relative protection in other diabetic models [58,59]. These findings suggest that oxidative and metabolic responses may differ by sex, but T1DM, cell, and genetically modified models remain indirect for the predominant clinical question in T2DM.

The direction of sex-related injury is not consistent across models: some show female susceptibility, others male susceptibility, and eNOS-deficient or uninephrectomized db/db models show similar albuminuria despite molecular differences [55,60,61]. Model, diabetes type, age, gonadal status, and the stage of kidney injury can all alter the observed phenotype. Molecular differences without a corresponding difference in albumin excretion also caution against equating pathway activation with clinically important effect modification. The most plausible pathways involve hormonal regulation, intrarenal hemodynamics, podocyte integrity, and tubular oxidative or metabolic responses, but none has been shown to account for a different cardiovascular risk associated with the same UACR in humans.

## 7. Sex- and Gender-Related Sources of Ascertainment and Research Bias

Sex and gender are analytically distinct. Sex is relevant to UACR calibration and biological effect modification; gender encompasses social roles, behaviors, identities, and structural conditions that may influence access to testing and treatment. Routine datasets usually record sex but rarely measure gender directly, so differences observed between women and men should not automatically be labeled gender effects [11,62,63]. Conversely, recording sex alone does not make an observed disparity biological. Interpretation requires attention to what was actually measured and to plausible health-system pathways.

UACR remains underused in diabetes care. In a U.S. analysis of 513,165 adults with T2DM, complete CKD assessment using both eGFR and UACR was uncommon [64]. European, U.S., and Australian studies further report lower albuminuria monitoring or less guideline-concordant CKD care among women [13,14,65,66]. These are disparities documented by sex; disease phenotype, contraindications, clinician behavior, social constraints, continuity of care, and health-system access may contribute, but their relative roles are not resolved. The available studies therefore support a problem in ascertainment and care, not a single explanation for it.

Differential testing can bias the evidence reviewed here. If women receive UACR measurement mainly after a complication or recognized high-risk feature, women with recorded UACR may be more selected than men. Conditioning an analysis on the availability of UACR can then change the distribution of comorbidity, kidney phenotype, and treatment in the analytic sample. In causal terms, measured UACR may act as a selection variable influenced by both health-care contact and clinical risk, creating or obscuring associations after stratification. This may alter UACR distributions, event rates, and apparent sex-specific associations in routine-care cohorts. The mechanism is plausible but has not been shown to explain the heterogeneous findings in Section 4.

Research design creates a second limitation. Women have been underrepresented relative to CKD prevalence in phase 3–4 trials, and efficacy or safety results are infrequently reported by sex [67]. Dialysis and critical-care nephrology trials similarly show limited sex- or gender-based analysis and sometimes poorly justified reproductive exclusions [68,69]. When women are included, but event numbers are low, sex-stratified estimates may be unstable and formal interaction tests underpowered. These practices reduce both external validity and the ability to distinguish genuine interaction from chance subgroup variation.

The SAGER framework recommends explicit and consistent reporting of sex and gender [70]. For the present question, reports should provide sex-specific estimates, confidence intervals, and event numbers together with a formal sex-by-UACR interaction; describe how and when UACR was measured; and report whether testing, missingness, exclusions, or imputation differed by sex. Analyses should also clarify whether sex was prespecified as an effect modifier and how multiplicity was handled. A significant estimate in one sex and a non-significant estimate in the other is not sufficient evidence that the effects differ.

These considerations are not peripheral to the central question: differential ascertainment determines who enters an observational analysis, whereas representation, event numbers, and statistical power determine whether a genuine interaction can be detected. They also affect transportability, because an interaction estimated among selectively tested patients may not apply to the wider T2DM population. Their principal implication is methodological caution and better reporting, not a biological explanation for separate UACR thresholds.

## 8. Focused Clinical and Therapeutic Implications

A graded association between UACR and cardiovascular risk below 30 mg/g is a prognostic observation, not a treatment indication. A possible difference in prognostic strength likewise does not imply that women and men require different drugs. A prognostic interaction asks whether the marker carries different information; a treatment interaction asks whether the benefit or harm of an intervention differs. These questions require different analyses and should not be inferred from one another. UACR should inform the overall cardiorenal profile, whereas therapy should follow the populations and indications tested in randomized trials and adopted in guidelines [1,2].

Current recommendations preserve this distinction. RAS blockade is indicated in diabetes, CKD with A2–A3 albuminuria, and other established indications; SGLT2 inhibitors, GLP-1 receptor agonists, and finerenone are used according to eGFR, CKD, heart failure, glycemic, and albuminuria criteria [1,2]. A confirmed UACR of 10–29 mg/g may identify higher risk than very low UACR, but in isolation it neither establishes CKD nor supports starting RAS blockade or finerenone. It may instead justify confirmation, closer assessment of the overall phenotype, and attention to therapies already indicated for diabetes, heart failure, or established CKD. ROADMAP illustrates why improvement in an albuminuria surrogate cannot automatically be equated with cardiovascular benefit [71].

Available randomized evidence does not support sex-specific prescribing. Dapagliflozin in DECLARE-TIMI 58 and canagliflozin in CREDENCE showed no clinically persuasive sex heterogeneity in cardiovascular or kidney effects [72,73]. A meta-analysis of GLP-1 receptor-agonist outcome trials found benefits in women and men without significant sex interactions for kidney outcomes or MACE [74]; greater weight loss in women in other trials should not be conflated with greater cardiorenal benefit [75]. FIDELITY likewise found broadly consistent finerenone effects by sex, although individual subgroup signals remain exploratory [76]. These analyses were generally secondary and may have limited power to detect modest heterogeneity. Their consistency supports common current indications, but absence of interaction should not be interpreted as proof of identical biological response.

Sex may influence the biomarker response itself. Female sex was associated with greater UACR lowering across randomized groups in post hoc CONFIDENCE analyses [77], while early UACR reduction predicted subsequent outcomes in LEADER, EMPA-REG OUTCOME, and finerenone mediation analyses [31,32,78]. A difference in percentage UACR change may reflect baseline distribution, adherence, hemodynamic response, body composition, or regression to the mean. Mediation analyses can support albuminuria as part of the treatment pathway but cannot establish it as the sole causal mechanism. These findings therefore do not identify a sex-specific drug choice or percentage-reduction target.

The practical priority is systematic measurement, confirmation of abnormal or borderline results, repeated standardized sampling when feasible, interpretation with eGFR and total cardiovascular risk, and equitable access to proven therapy. Real-world data suggest that women with CKD receive some guideline-directed therapies less often [65,66,79,80]. Auditing testing and prescribing by sex may identify remediable care gaps, but any intervention should address the demonstrated barrier rather than assume a biological difference. Sex can add context to risk interpretation, but it should not override trial-based treatment indications.

## 9. Knowledge Gaps and Future Directions

Future cohorts should prespecify sex-by-UACR interactions, include enough participants and events to test them, and report sex-specific estimates with confidence intervals and interaction terms. Power calculations should be based on the interaction rather than on the main UACR association, because subgroup comparisons require substantially larger event numbers. Within A1, UACR should be modeled continuously with flexible methods such as restricted cubic splines rather than divided into post hoc sex-specific categories. Hard cardiovascular outcomes should be separated from arterial stiffness, myocardial fibrosis, and other surrogate phenotypes because outcome-specific patterns cannot be assumed to represent a universal effect [36,37,38,40,44]. Pre-specification and careful control of multiplicity are essential when several outcomes, UACR forms, and subgroups are examined.

Repeated measurement is essential. Biological variability is substantial at low UACR and may differ by sex [35,52]. Li et al. identified four UACR trajectories in 601 people with T2DM and found greater MACE risk with persistently high and increasing patterns [81]. Chen et al. studied 3101 people with T2DM and baseline UACR < 30 mg/g; sex was associated with trajectory membership, while mid-range and rising-with-fluctuation patterns predicted progression to proteinuria [82]. These studies show the value of longitudinal phenotyping, but neither established sex modification of hard cardiovascular outcomes. Prospective studies should use repeated first-morning samples and capture persistence, progression, regression, intermittent increases, and cumulative exposure.

The ratio should also be unpacked. Studies should report urinary albumin and creatinine separately and include body composition, age, and metabolic health; timed or 24-h albumin excretion in a validation subset could distinguish ratio calibration from absolute albumin loss [45,46,47,48,49]. Body-composition measures are especially relevant in older adults, obesity, frailty, and during treatments that change weight or muscle mass. Hormonal context should be measured rather than inferred from age: prospective datasets should record menopausal status, age at menopause, hysterectomy or oophorectomy when relevant, and menopausal hormone therapy. Mechanistic substudies could add estradiol, testosterone, and sex hormone-binding globulin, while recognizing that observational associations cannot establish causality. T1DM and experimental findings should be analyzed separately from T2DM and identified as indirect when used to support mechanisms [16,17,18,54,55,56,57,58,59,83].

Treatment exposure requires longitudinal modeling because RAS blockade, SGLT2 inhibitors, GLP-1 receptor agonists, and finerenone alter UACR and cardiorenal risk. Initiation, discontinuation, adherence, dose changes, and combination therapy should be captured so that spontaneous change is not confused with treatment-associated response [31,32,77,78]. Time-varying confounding is likely, as treatment is both influenced by kidney phenotype and capable of changing subsequent UACR and risk.

A statistically reproducible interaction would still be insufficient for clinical adoption. The key test is whether adding a sex-by-UACR term improves calibration, discrimination, reclassification, decision curves, or treatment decisions beyond a model containing sex and UACR as separate predictors. Tao et al. showed that UACR improved heart-failure prediction in T2DM with broadly consistent associations across sex subgroups [84]; whether an interaction term adds clinically useful information remains unknown. Any improvement should be reported with confidence intervals, compared with established clinical models, and validated in an independent population rather than judged from statistical significance alone.

External validation should span ages, ancestries, health-care systems, diabetes types, kidney-function ranges, and cardiovascular phenotypes. Studies using electronic health records should state how many eligible participants had UACR available and whether missingness differed by sex [13,14,62,63,65,66,67,68,69,70]. An informative prospective cohort would combine repeated first-morning UACR, separate albumin and creatinine measures, body composition, reproductive and hormonal variables, detailed cardiovascular phenotyping, and time-varying treatment. It would prespecify adequately powered interactions and test whether they improve prediction or decisions. Until a robust interaction meets those criteria, sex should inform study design and clinical context but not separate diagnostic or therapeutic thresholds.

## 10. Conclusions

UACR predicts cardiovascular risk in women and men with diabetes across its distribution, including within A1. The evidence that sex modifies this relationship is much weaker: formal interactions are limited to selected populations and outcomes, direct evidence within A1 is sparse, and several apparent differences arise only from sex-stratified analyses. Heterogeneity may reflect outcome definition, population, ratio calibration, kidney biology, sampling, treatment, selection, or statistical power. It does not establish a consistent direction of effect.

Clinical care should use UACR systematically as a continuous risk marker while preserving validated diagnostic and therapeutic thresholds. Abnormal or borderline values should be confirmed and preferably repeated under standardized conditions; results should be interpreted with eGFR, cardiovascular phenotype, and the overall risk profile. Sex may provide additional context, but current evidence does not support sex-specific UACR cut-offs, treatment indications, or albuminuria-response targets.

## Figures and Tables

**Table 1:** Hierarchy and analytical interpretation of key studies evaluating sex differences in albuminuria-associated cardiovascular outcomes.

Study/Population and Evidence Scope	Design/Follow-Up	UACR Range and Endpoint	Sex-Stratified Finding	Formal Interaction Status
Nitsch et al. (2013) [34] General/CKD; indirect	Individual-participant meta-analysis of 46 cohorts; follow-up varied	Broad albuminuria range; all-cause and cardiovascular mortality	Risk increased in both sexes; relative gradient steeper in women	Significant formal sex interaction
Hatlen et al. (2016) [35] General population; indirect	Prospective cohort; 14 years; three urine samples	Repeated UACR; MI or coronary death	Similar association in women and men	Formal comparison not significant (*p* = 0.3)
Dal Canto et al. (2023) [36] Diabetes (98.9% T2DM); direct for HF, not A1-specific	Prospective cohort with annual measures; mean 7 years	UACR > 3 mg/mmol (≈27 mg/g) vs. lower; MI, CHD, stroke, HF, cardiovascular mortality	Higher HF risk in women but not men; other endpoints did not show this pattern	Significant formal interaction for HF only (*p* = 0.010)
Siverio-Morales et al. (2025) [37] T2DM and A1; direct	Retrospective cohort; 6 years	UACR < 30 mg/g; coronary stenosis and MACE	Associations in both sexes; estimates directionally stronger in women	Formal UACR-by-sex interaction not significant
Xuan et al. (2026) [38] T2DM; indirect surrogate evidence	Cross-sectional; no follow-up	Normal-range UACR; baPWV	Association reported in women, not men; postmenopausal signal	Sex-stratified result; no formal interaction reported
Pattar et al. (2025) [39] CKD; indirect surrogate evidence	Small cross-sectional study; no follow-up	UACR; AIx, pulse wave velocity, mean arterial pressure	Positive UACR-AIx association in women, not men	Formal interaction for AIx; no interaction for other vascular measures
Tran et al. (2026) [40] Healthy older adults; indirect	Prospective ASPREE cohort; median 8.3–8.6 years	Low-level UACR; mortality, CVD, MACE, stroke	Mortality association in both sexes; cardiovascular associations more apparent in men	Sex-stratified result; no formal interaction reported

Abbreviations: AIx, augmentation index; baPWV, brachial-ankle pulse wave velocity; CHD, coronary heart disease; CKD, chronic kidney disease; CVD, cardiovascular disease; HF, heart failure; MACE, major adverse cardiovascular events; MI, myocardial infarction; T2DM, type 2 diabetes mellitus; UACR, urinary albumin-to-creatinine ratio. Direct evidence denotes a diabetes-specific population and an outcome relevant to the review question; surrogate outcomes and non-diabetic populations are classified as indirect. Sex-stratified significance alone is not evidence of interaction.

**Table 2:** Factors potentially influencing the measurement and cardiovascular interpretation of UACR in women and men.

Domain	Sex-Related Biological or Gender/Health-System Factor	Potential Consequence for UACR or Its Interpretation	Clinical/Research Implication
Measurement	Lower urinary creatinine excretion in many women	Higher UACR for the same absolute albumin excretion.	Interpret UACR alongside age and body composition; avoid assuming identical absolute albumin excretion.
	Greater fat-free mass and urinary creatinine excretion in some men	A normal or lower UACR may coexist with elevated absolute urinary albumin excretion.	Consider denominator-related underestimation when UACR and the clinical phenotype appear discordant.
	Within-person biological variability, particularly at low UACR	Misclassification near category thresholds and unstable risk estimates.	Confirm abnormal or borderline findings with repeated measurements; prefer first-morning samples when feasible.
	Spot versus first-morning or timed/24-h urine collection	Potential discordance between UACR and absolute urinary albumin excretion.	Report the sampling strategy and use consistent protocols in longitudinal studies.
Biology	Sex hormones and reproductive status	May modify podocyte survival, glomerular injury, and the context in which albuminuria develops.	Measure menopausal and hormonal context where relevant; this plausibility does not establish different clinical thresholds.
	Sex-dependent RAS and intrarenal hemodynamics	May translate similar metabolic stress into different patterns of albuminuria and structural injury.	Interpret experimental findings cautiously and test phenotype-specific associations in humans.
	Tubular oxidative, metabolic, and epigenetic responses	May generate sex-related susceptibility or protection during hyperglycemia.	Integrate sex, hormonal exposure, and cell-intrinsic pathways in translational studies.
Gender/health-care and research	Lower albuminuria-testing rates among women in several health-care settings	Differential ascertainment and selection into datasets with available UACR	Report testing frequency and missingness by sex; examine plausible gender and health-system pathways
	Underrepresentation and limited sex-disaggregated reporting	Low power for genuine sex-by-UACR interactions	Prespecify interactions and distinguish them from sex-stratified associations
Treatment	RAS blockade, SGLT2 inhibitors, GLP-1 receptor agonists, and finerenone	Can modify UACR and cardiorenal risk over time, potentially changing observed associations.	Model treatment exposure longitudinally and distinguish spontaneous UACR change from treatment-associated response.

Abbreviations: GLP-1, glucagon-like peptide-1; RAS, renin–angiotensin system; SGLT2, sodium-glucose cotransporter 2; UACR, urinary albumin-to-creatinine ratio.

## Data Availability

No new data were created or analyzed in this study. Data sharing is not applicable to this article.
